# Comment on ‘Tissue-infiltrating immune cells as prognostic markers in oral squamous cell carcinoma: a systematic review and meta-analysis’

**DOI:** 10.1038/s41416-019-0459-9

**Published:** 2019-04-23

**Authors:** Lu Zhou, Ming Li, Tao Zhu

**Affiliations:** Department of Anesthesiology and Translation Neuroscience Center, West China Hospital, Sichuan University, Chengdu, Sichuan China

**Keywords:** Tumour biomarkers, Oral cancer

We read with great interest the recently published systematic review and meta-analysis by Olsen et al.^1^ entitled ‘Tissue-infiltrating immune cells as prognostic markers in oral squamous cell carcinoma: a systematic review and meta-analysis'. This work contributes the knowledge to the area regarding significant prognostic value of tissue-infiltrating immune cells in oral cancer. The authors concluded that CD163+ M2 macrophages and CD57+ natural killer cells may be the most promising predictors of survival in oral cancer patients. We appreciate the authors’ thorough analysis, which is a clinically valuable study. However, several important methodological issues should be noted, which may influence the strength of their conclusion in this review.

First, the authors limited their search to Medline and Embase, and did not explore other international databases with broad subject areas, including Cochrane Library, ClinicalTrials.gov and Web of Science databases. Selection of a restricted subset of databases for conducting the literature search can lead to biased results, even incorrect conclusions.^2^

Second, Olsen et al.^1^ did not provide the relevant methodology on how they assessed the publication bias in this review. Lack of publication bias assessment may exaggerate the correlation between predictors and survival of oral cancer patients.^2^ The necessity of evaluating publication bias and its role in providing the best evidence has been established in meta-analysis.^3^

Finally, although the authors assessed the quality of the included studies using the REMARK guidelines, we could not tell that the risk of bias of the included studies was high, medium or low. Other methods for assessment of publication quality and risk of bias may be more suitable, such as the Quality in Prognostic Studies (QUIPS) tool^4^ or the Newcastle-Ottawa Scale (NOS).^5^ In addition, it would be better if the authors could provide a quality grading of the evidence in this review.

Overall, the authors analysed a valuable issue regarding the prognostic markers in oral squamous cell carcinoma, but the results of this review should be interpreted with caution due to the limitations mentioned above. We believe that these methodological concerns of this meta-analysis are likely to raise questions about interpretation of the authors’ findings and their clinical relevance.
